# Factors Affecting the Tissue Damaging Consequences of Viral Infections

**DOI:** 10.3389/fmicb.2019.02314

**Published:** 2019-10-04

**Authors:** Deepak Sumbria, Engin Berber, Barry T. Rouse

**Affiliations:** Department of Biomedical and Diagnostic Sciences, College of Veterinary Medicine, The University of Tennessee, Knoxville, Knoxville, TN, United States

**Keywords:** viral infection, immunopathology, outcome of past and concomitant infections, influence of microbiome and virome, therapeutic management of infections

## Abstract

Humans and animals are infected by multiple endogenous and exogenous viruses but few agents cause overt tissue damage. We review the circumstances which favor overt disease expression. These can include intrinsic virulence of the agent, new agents acquired from heterologous species, the circumstances of infection such as dose and route, current infection with other agents which includes the composition of the microbiome at mucosal and other sites, past history of exposure to other infections as well as the immune status of the host. We also briefly discuss promising therapeutic strategies that can expand immune response patterns that minimize tissue damaging responses to viral infections.

## Introduction

Humans are perpetually infected with multiple endogenous and exogenous viral agents and estimates suggest that some of us generate up to 10^12^ new virus particles per day (Virgin et al., 2009). Moreover, thanks to astonishing advances in technology we are constantly discovering new viruses that are present somewhere on or in our bodies. However, few exogenous viruses cause overt disease and when this does occur usually only a minority of individuals are affected. Since viruses are obligate intracellular parasites that require host cells to replicate and assemble infectious progeny, the question arises as to the nature of factors which impact on whether or not infections are unobserved or develop into some form of overt cell and tissue damage. Some viruses do have high intrinsic levels of pathogenicity and mediate notable, often lethal, tissue damage in a high proportion of infected individuals. Smallpox was the poster child of this situation in mankind. These days, Ebola virus and some other viruses that cause hemorrhagic fevers have high intrinsic pathogenicity causing dramatic effects that include death in most infected persons (Feldmann and Geisbert, 2011).

In animal populations, highly virulent viruses also occur from time to time. For example, when it first occurred in the 1950s, myxomatosis killed more than 95% of the rabbit population in parts of Europe and Australia (Kerr, 2012). Every so often new virulent strains of influenza (Flu) appear after reformulation usually in birds or swine. These variants can be devastating to humans, as happened in 1918–1919 and often occurs with concentrated poultry flocks (Taubenberger and Morens, 2006; Peiris et al., 2007). This has occurred a few times in southern China and was of great concern when strain H5N1 came onto the scene since spread to humans was anticipated (Peiris et al., 2007). The likelihood that any virus infection can cause disease is increased if the host has a clear-cut defect in one or more components responsible for immune protection, but this situation is uncommon. A more common cause of tissue damage is that the circumstances of infection result in an imbalanced host response with one or more components that are proinflammatory dominating over regulatory activities. In these situations, the host response to the infection, which in many instances is persistent and difficult to remove, is the main cause of tissue damage. Most commonly the main cellular orchestrators of such lesions are certain subsets of T cells with antigen non-specific inflammatory cells and various mediators causing the actual tissue damage (Rouse and Sehrawat, 2010).

## Exotic Infections Can Be Highly Tissue Damaging

Several circumstances during a viral infection can set the stage for tissue damage (Belkaid and Rouse, 2005; Rouse and Sehrawat, 2010). Foremost of these is infection by an exotic agent invariably derived from another species where the virus may be an asymptomatic natural infection. Indeed, we believe that many of our common virus infections were originally introduced at the time when human populations came to live more closely together and along with animals. This happened when many humans became agriculturalists rather than being more solitary hunters and gatherers. Several infections were acquired from their newly domesticated livestock such as measles, likely derived from rinderpest infected cattle and sheep (Pearce-Duvet, 2006). However, it is surmised that most human virus pathogens were acquired from rodents (>60) and about the same number from bats (Luis et al., 2013). We are still acquiring exotic infections particularly from other primates, and when this occurs they are often clinically severe and sometimes lethal. Most famous of these exotic infections was the occurrence of AIDS in the early 1980s now known to be caused by human immunodeficiency virus (HIV) derived from a non-human primate (Sharp and Hahn, 2011). More recently primate derived Ebola and Zika viruses have been a cause for concern and bat derived Nipah virus is a frequent problem in Bangladesh (Faria et al., 2016; Malvy et al., 2019; Nikolay et al., 2019). The dog communities received a pathogenic gift from cats-parvovirus which caused major clinical problems worldwide, but now well controlled by vaccines (Parrish et al., 1985). The aforementioned myxomatosis virus which killed so many European rabbits was originally a natural well tolerated pathogen in Brazilian wild rabbits (Arthur and Louzis, 1988). The current devastating epidemic of African swine fever virus in Chinese pigs is an inapparent infection in its original host-warthogs and bush pigs in Africa (Meng et al., 2009).

Whereas exotic infections can cause severe clinical consequences when they first emerge, in most instances the virus-host relationship undergoes genetic changes on both sides and the interaction evolves to become less tissue damaging. This happened remarkably quickly with myxomatosis in the fast breeding rabbit population. Adaptation also occurs successfully if the exotic agent induces protective immunity as happened with the flaviviruses Zika and yellow fever. In contrast, adaptation is prolonged with agents that induce inferior immunity such as with the flavivirus hepatitis C virus (HCV). Exotic virus infections which fail to induce protective immunity and at the same time may be immunosuppressive, such as HIV, are proving difficult to control with vaccines.

## Infection by Viruses That Blunt Components of Immunity

Another situation which sets the stage for tissue damage is when the virus has properties that blunt the efficacy of one or more components of host defense. This immune blunting property, more commonly referred to as immune evasion, may also impact on the response to heterologous infections as is discussed in a subsequence section (the influence of previous and concomitant infections). The topic of immune evasion has received intense study and has received many excellent reviews (Ploegh, 1998; Alcami and Koszinowski, 2000). Blunting the efficacy of protective immunity is particularly consequential with HIV, but all members of the herpesvirus family possess strategies that interfere with effective host immunity (Kumar et al., 2018). In their natural hosts, herpesviruses usually persist, resist immune removal and usually cause minimal disturbance in immunocompetent hosts (Ploegh, 1998; Sehrawat et al., 2018). The most genetically complex of all herpesviruses, human cytomegalovirus (HCMV), has an abundance of immune blunting activities and seldom causes relevant tissue damage in immunocompetent hosts (Nolan et al., 2017; Picarda and Benedict, 2018). Like all herpesviruses, HCMV can establish an alternative replication pattern called latency where the virus can persist for prolonged times and may make them invisible to the immune system (Picarda and Benedict, 2018). This is the state of affairs with herpes simplex virus (HSV), but with this herpesvirus periodic recurrences often result in tissue damaging episodes which can be more of consequence when the immune system is dysfunctional or unbalanced (Van de Perre et al., 2008). In part because of the many ways by which herpesviruses avoid and manage host immune responses, we have few effective vaccines and antivirals against these agents.

## Influence of Age at Time of Infection

Whether or not a virus infection causes significant tissue damage often depends on the age at which the infection occurs. Generally speaking, young children and elderly people are more susceptible to the tissue damaging consequences of virus infection than healthy adults. For instance, several respiratory viral infections such as Flu and respiratory syncytial virus (RSV) and some gastrointestinal infections cause more severe disease in young persons, particularly infants (Collins and Graham, 2008). Old individuals can also suffer more severe problems with Flu as well as with RSV, especially if they reside in community centers. Some infections are particularly damaging if they infect the fetus especially in those species that transfer minimal or no maternal antibody across the placenta during pregnancy. Thus, *in utero* infection of non-antibody protected calves with bovine diarrhea virus or bovine herpesvirus can either result in abortion or congenital abnormalities (Baker, 1995). A similar outcome can occur in foals when infected *in utero* with equine herpes virus and lambs infected *in utero* with bluetongue virus (Clarke and Osburn, 1978; Walter et al., 2013). The recent global concern that happened when Zika virus first appeared in Brazil causing congenital and other problems has now receded (Faria et al., 2016). Thus, Zika has become endemic and many women are infected and develop protective immunity before they become pregnant. Moreover, other flaviviruses also cross-react with Zika and these may also confer cross-protection or potentially could increase pathogenicity (Stettler et al., 2016).

The situation with Zika recalls the past consequences of maternal infection with rubella in the pre-vaccination era. Thus, if seronegative women were infected with rubella during pregnancy their children could have a range of congenital lesions that often involved the nervous system (Bouthry et al., 2014). Today HCMV infection remains a problem if infection occurs *in utero* or around the time of birth. Children can develop congenital CMV, a syndrome that mainly affects the nervous system. The most common problem is hearing loss, but problems with vision and cognition also occur (Schleiss, 2018). A small minority develop microcephaly the major sign noticed when Zika first occurred. Curiously, infants can develop congenital CMV when born to seropositive mothers (Britt, 2018) since adaptive immunity does not preclude herpesvirus infection–bad news for vaccinologists.

The observation that young animals infected with some viruses can develop more tissue damaging responses than do adults can have multiple explanations. For instance, several aspects of the immune system are not fully functional *in utero* and even after birth. For example, the type-I interferon response and the activity of some subsets of dendritic cells (DC) are less active, the specific immune repertoire may be lacking some reactivities, the immune reactivity pattern lacks regulatory components and many signaling pathways may be not fully functional. This topic has been reviewed by others (Samuel, 2001; García-Sastre, 2002; Katze et al., 2002). Older animals too may develop more severe responses to virus infections because of senescence changes to the immune system. This subject will not be further discussed but is under intense investigation with many excellent reviews available (Nikolich-Žugich, 2008; Maue et al., 2009).

Finally, of some interest is that some virus infections that persist and cause no tissue damage for decades may suddenly become very consequential. This may happen with varicella zoster virus (VZV) which years after causing chicken pox in children (or even vaccination against chickenpox) persons may develop distressing and painful dermal lesions called shingles (John and Canaday, 2017). This represents reactivation from VZV latency and it usually occurs in only one or a few ganglia of the far greater number of ganglia that are latently infected. The lesions represent T cell-mediated immunopathological reactions, and ultimately the recurrent infection is controlled by the immune system. Among the unsolved mysteries of shingles are explaining the circumstances of its occurrence, why only a minority of the many infected ganglia reactivate and why a vaccine that largely induces only antibody-mediated immunity appears to be so efficacious to prevent shingles.

## The Influence of Dose and Route of Infection

Several additional circumstances during viral infection can influence the outcome in terms of being subclinical or tissue damaging. Exposure dose and route of infection are relevant variables. In fact, it is likely that the majority of persons who escape clinical infections during Flu outbreaks could in part be explained by a lifestyle that limits the level of infection. Those of us who have few close interactions with others, and especially if we adopt a non-affectionate behavior pattern, are less likely to receive high dose infections. However, quantitative effects of infection can only be assessed by control studies in experimental animals. Influenza virus infections in mice have been used for this purpose (Legge and Braciale, 2005). In such studies, animals given a high dose all developed disease and this could be lethal. The deaths seemed to be the consequence of the otherwise protective CD8 T cell response being suppressed. The suppression was explained by plasmacytoid DC, which express Fas-ligand upon Flu infection, making contact with Fas-expressing CD8 effector T cells causing these otherwise protective cells to undergo apoptosis. A similar form of plasmacytoid DC/leucocyte interaction could explain the lymphopenia which occurs with virulent H5N1 flu infection (Langlois and Legge, 2010). At lower doses of flu infection, other types of DC were preferentially infected but these cells fail to express Fas-ligand permitting protective T cells to survive. At even lower doses of virus one can assume the virus is fully controlled by innate defenses and adaptive aspects of immunity may not be induced.

A few years back when infection experiments in chimpanzees were still permitted, some remarkable observations on the infection dose effects was reported by the Chisari group (Asabe et al., 2009). They showed that when animals were infected with between 10^4^ to 10^8^ virions of hepatitis B virus, animals developed mild hepatitis and readily controlled the disease. However, infection at a higher dose resulted in damaging chronic active hepatitis thought to be an immuno-pathological response to the infection. Surprisingly, infecting chimpanzees with a minimal dose which could be as low as 100 virions gave the same outcome as those infected with greater than 10^8^ virions. Explanations for these observations were not at hand but it could have related to the virus avoiding contact and elimination by some aspect of innate immunity, but still able to present antigens to induce an unbalanced T cell response that mediated tissue damage.

Apart from dose effects of infection, the route of exposure can also be critical. With HIV, for example, infection is more likely to occur when virus infects the rectal compared to the vaginal epithelium (Tebit et al., 2012). With experimental HSV infections in mice, route critically influences consequences. Thus, if animals are infected subcutaneously or intramuscularly, the outcome is usually subclinical and a full range of adaptive immune responses are induced. However, intradermal infection on the flank gives rise to a so-called zosteriform reaction in the whole dermatome which innervates the site of infection (Simmons and Nash, 1984). These lesions resemble those which occur in human shingles. The skin lesions represent host immunopathological reactions to virus reemerging at nerve endings after first replicating in the innervating nerve ganglion. Similar immunopathological lesions occur in the eye when virus gains access to the cornea. This lesion is referred to as stromal keratitis and it is a common cause of vision impairment in humans (Pepose et al., 1996). Curiously, if HSV is delivered to the anterior chamber of the eye some aspects of adaptive immunity are induced but not others. The consequences of anterior chamber administration may be retinitis which occurs in the contralateral but not in the ipsilateral retina which is thought to be protected by an interferon response (Vann and Atherton, 1991).

Infection by the mucosal route often results in minimal tissue damage, in part because infection by this route preferentially induces regulatory T cells and cytokine responses such as IL-10 producing macrophages (Rezende and Weiner, 2017). This induction of oral or nasal tolerance is usually accomplished using soluble antigens at a critical dose level and although viruses when given orally were suspected to induce a form of tolerance but because most infections induce an inflamed environment the idea is no longer in favor (Rezende and Weiner, 2017). Some enteric virus infections, however, can terminate oral tolerance induced against autoantigens and allergens likely by establishing a more inflammatory less regulatory immune environment (Rezende and Weiner, 2017).

## Influence of Host Genetics

The outcome of a virus infection can be profoundly influenced by host genetics especially by genes that encode major innate or adaptive components of immunity. Thus, animals lacking the ability to generate T lymphocytes become highly susceptible to many virus infections (Dropulic and Cohen, 2011). Some infections that are usually inconsequential can be severe or even lethal in those with T cell defects. Infections by herpesviruses illustrate this scenario (Ruffner et al., 2017). By way of contrast, genetic defects which affect antibody production usually result in more clinical problems with bacterial than viral infections (Carneiro-Sampaio and Coutinho, 2007). Humans have approximately 23,000 genes and many of these can influence the efficacy of virus control. However, usually the deletion or changed function of a single gene product has only a partial effect on responses and then only under certain circumstances of infection. For example, the phenotype of the chemokine receptor gene CCR-5 may be associated with greater resistance to HIV infection, but this resistance is readily overcome at higher doses of HIV exposure (Wilkin and Gulick, 2012). Curiously, the same phenotype of CCR-5 that increases resistance to HIV renders persons more susceptible to West Nile virus (Glass et al., 2006).

In addition to CCR-5, many other genes that influence innate immune function can impact on the severity of virus infections. For example, several genes that affect interferon or interferon receptor functions can also result in more severe responses to several virus infections. Well studied examples are defects in genes such as toll like receptor (TLR) 3 and UNC93B (Casrouge et al., 2006). Both defects result in defective cytokine production especially interferon alpha and beta in response to infection. If babies with the defects are infected with HSV they can readily develop herpes simplex encephalitis (Casrouge et al., 2006). Defects in natural killer (NK) cell function may also result in more severe herpesvirus infections in humans (Biron et al., 1989; Orange, 2002). Countless functional defects in innate as well as adaptive immune activity have been produced in mouse models and many have been studied for effects on susceptibility to a wide range of viral infections. For example, studies on the pathogenesis and immunology of dengue and Zika virus in mice only became possible using mice when the interferon receptor was knocked out (IFN-R^–/–^) making them susceptible to infection (Lazear et al., 2016).

## Influence of the Microbiome

Whereas dysfunctions or deletions of host genes can affect the efficacy of antiviral defense, the far more abundant genetic material present in the form of microbes acting as passengers within or on the body surfaces may also influence the outcome of infection by a pathogen. The focus of attention has been on the microbiome of the gut and skin whose combined genetic material can exceed 10^13^ gene and hence far exceeds the number of host genes (Bik et al., 2006). The microbiome, particularly of the gut, impacts on the disease pattern of numerous syndromes. Many excellent reviews have been written and we shall comment only briefly emphasizing effects of the microbiome on responses to virus infections (Honda and Littman, 2012; Belkaid and Harrison, 2017). The microbiome consists of several families of bacteria (10^11^ per gram/contents), fungi, viruses, eukaryotes such as the protozoan Blastocystis as well as helminths. The actual composition of the microbiome differs during development and is strongly influenced by diet, previous exposure to infections and other factors (Honda and Littman, 2012; Thomas et al., 2017). Evidence accumulates to show that the composition of the microbiome can markedly affect how the host responds to exogenous viral pathogens. Most studies have measured the effects of the bacterial microbiome. If this is diminished by treatment with broad spectrum antibiotics, or removed by germ free procedures, responses to several viral infections are changed. Overall, the manipulated animals generate more tissue damaging responses to several infections compared to controls. One of the first to be studied was lymphocytic choriomeningitis virus (LCMV) which becomes more severe or prolonged in antibiotic purged mice (Abt et al., 2012). The outcome was explained by a reduced antibody and CD8 T cell response in treated mice perhaps associated with diminished TLR induced cytokine and chemokine responses involved in immune induction (Abt et al., 2012). Influenza virus infections can also be more severe in antibiotic treated animals because of increased viral replication in lungs perhaps mainly a consequence of a reduced inflammasome response in treated animals which led in turn to reduced T cell priming (Ichinohe et al., 2011). Experimental flavivirus infections in IFN-R^–/–^ mice may also be more severe in antibiotic treated mice, an effect explained also by a reduced innate and adaptive response to the infection (Thackray et al., 2018).

Antibiotic purging can also cause some virus infections to become less tissue damaging. This pattern of events can occur with mouse rotavirus and norovirus infection (Uchiyama et al., 2014; Baldridge et al., 2015). The subject is currently of high interest since it has become evident that the resident microbiome may influence how many viruses are controlled and how well persons respond to vaccines. For example, the lesser effectiveness of rotavirus vaccines in some developing countries could be explained by the composition of their microbiome.

The mechanisms by which the resident microbiome could influence the outcome of other disease syndromes is under intense investigation. Ideas include effects on the expression or access of cell receptors required for viral entry, the production of microbial products that bind to viruses and influence their infectability and, perhaps of most relevance, the effect the microbiome has on the pattern of immune responsiveness to both mucosal and systemic infections. As regards effects on viral receptors one study showed that the bacterial product flagellin affected susceptibility to rotavirus and to a lesser extent reovirus infection (Uchiyama et al., 2014). The flagellin was shown to act via TLR5 and nucleotide oligomerization domain (NOD)-like receptor 4 engagement to induce IL-22 and IL-18 which likely caused apoptosis of the target cells that normally support virus replication. Thus, pre-exposure of mice to flagellin could prevent rotavirus infection and even accelerate cure of established infections. Additionally, the observation that the presence of *Staphylococcus aureus* in the lungs of mice prior to Flu infection resulted in reduced mortality was explained by changing TLR2 induced recruitment of inflammatory cells which inhibited the Flu infection (Wang et al., 2013; Zhang et al., 2014).

An example of the effect of a microbial product on the efficiency of infection by a virus was observed with poliovirus where optimal infectivity was observed to depend on the virus binding to bacterial surface polysaccharides and peptidoglycans (Kuss et al., 2011; Robinson et al., 2014). The bound virus was also more stable which facilitated transmission. Human norovirus infections also become more infectious when bound to bacterial surface glycans (Norman et al., 2014). Another example is vertical infection with mouse mammary tumor virus. Here the binding of bacterial lipopolysaccharides (LPS) to the maternal-derived virus caused the production of immunosuppressive IL-10 in the pups and this facilitated their infection (Kane et al., 2011). The microbiome might also play a role in dengue virus (DENV) infection. Here too LPS can enhance disease acting by increasing the production of platelet activating factor (PAF), TNF-alpha and IL-6 from DENV infected monocyte derived macrophages which worsened disease expression (Kamaladasa et al., 2016; Malavige and Ogg, 2017). The outcome occurred in part by PAF mediating increased vascular leakage, a significant component of dengue lesions (Kamaladasa et al., 2016).

Products from the microbiome can also help protect against the pathogenic effects of virus infections. For example, factors released from the microbiome residents *Lactobacillus casei* and *Bacteroides thetaiotaomicron* can bind to cell surface glycans and preclude infection by rotaviruses (Varyukhina et al., 2012). Indeed, rotavirus-associated diarrhea can be reduced by feeding Lactobacillus (Guandalini et al., 2000). Another recently reported example where microbiome-derived products decreased viral susceptibility was with herpes simplex encephalitis where a polysaccharide derived from the gut resident *Bacteroides fragilis* induced IL-10 producing protective B and T cells (Ramakrishna et al., 2019).

It is likely that the most common mechanistic explanation for the influence of the microbiome on the outcome of virus infections involve effects on the induction and activity of both innate and adaptive immune responses. The balance of microbiome constituents is in turn affected by many factors which include diet, stage of development and antibiotic use (Belkaid and Harrison, 2017). It is also curious to note that the gut microbiome of children born by natural delivery compared to caesarian section can show marked differences and this could influence their responses to viral infections (Mueller et al., 2015). With regard to dietary effects, a diet rich in fiber causes the production of short-chain fatty acids (SCFA) such as acetate, butyrate and propionate which favors the expansion of regulatory T cells in the mucosa and as well as systemically (Smith et al., 2013). This would provide an anti-inflammatory environment. On the contrary, diets low in fiber, such as we prefer in the United States, favor the expansion of segmented filamentous bacteria such as Clostridia species. These bacteria set the scene for a proinflammatory T cell responses, especially induction of the Th17 subset (Atarashi et al., 2011; Honda and Littman, 2012). The effect of diet on the outcome in terms of susceptibility to virus infection is under active investigation. Studies have revealed that Flu susceptibility can be affected by diet, although it is not clear if such effects proceed by affecting the composition of the microbiota. For example, mice fed a high fat diet led to a change in the balance of several immune parameters to favor inflammatory components (Milner et al., 2015). High fat recipients suffered more lung epithelial cell damage because of increased inflammatory cytokine (TNF-a, IL-6, INF-gamma, and IL-1 beta) responses and reduced regulatory T cells, M2 macrophages as well as decreased numbers of NK cells. Moreover, death rates were higher than animals fed a normal chow diet (Honce and Schultz-Cherry, 2019). High fat diets can result in obesity and there is also some epidemiological evidence that obesity could increase human susceptibility to Flu although the mechanisms involved are poorly understood (Gill et al., 2010). Conceivably, the observation that obese individuals had reduced ability to activate and perform functional CD4 and CD8 T cell responses as compared to healthy individuals could be one explanation (Paich et al., 2013).

Another way to evaluate microbiome effects on susceptibility to virus infection is to manipulate the diet in some way. This approach has received minimal study in humans and livestock and when any effects were observed they were not related to microbiota changes. The topic is certainly well worth further investigation. Thus, it would be curious to explain why the high consumption of fruits and vegetables seems to reduce the risk of upper respiratory viral infections in pregnant women (Li and Werler, 2010) and how feeding high levels of soya beans to pigs lowers their susceptibility to the important disease reproductive and respiratory porcine viral syndrome (Rochell et al., 2015). More diet manipulation studies have been done in experimental animals such as mice. For example, in a recent study a diet rich in the fermentable fiber inulin protected against Flu-induced pathology (Trompette et al., 2018). The mechanism proposed involved *Bacteroidetes* present in the gut which fermented the dietary inulin to produce SCFA. These SCFA were advocated to enhance protective CD8 T cell effectors and to reduce lung inflammatory effects by favoring M2 macrophages and reducing neutrophils (Trompette et al., 2018).

The bacterial microbiome is not the only component that can affect the outcome of virus infections. Thus enteric helminths infection can increase susceptibility to norovirus infection acting by inhibiting the protective CD8 T cell response (Osborne et al., 2014). Moreover, helminths are well known to influence patterns of immune responsiveness toward the Th2 pattern (Reese et al., 2014). This likely explains how helminth infection via inducing IL-4 and IL-13 can causes the breakdown of latency with some herpesviruses (Reese et al., 2014). Helminth infection might also impact on the severity of childhood RSV infection when the tissue damage is associated with a type II inflammatory response (McFarlane et al., 2017).

## Influence of the Virome

There is mounting evidence that other viruses which constitute the so-called virome also influence the consequences of pathogenic virus infections. Thanks to advances in sequencing and analysis of meta-genomic data many new viruses have been discovered especially in the intestinal tract. The great majority of these viruses are DNA bacteriophages and these may reach 10^15^ in number (Carding et al., 2017). Many of the rest are RNA containing plant viruses and about 1% are many types of passenger animal viruses that belong mainly to the anellovirus, parvovirus, adenovirus and papilloma virus groups (Neil and Cadwell, 2018). Added to the virome are the endogenous retroviruses which can account for up to 8% of the human genome as well as several DNA and RNA viruses in the body which act as chronic, but usually non-disease causing infections. Several of these latter agents belong to the herpesvirus family all of which can persist in the host in a non-replicating state referred to as latency.

It is strongly suspected that many components of the virome could influence the nature of the host response made to infection by an exogenous potential pathogen. For example, persistent infection with a non-pathogenic norovirus can reduce the extent of CD8 T cell responses which acts as major defense mechanisms against several viruses (Tomov et al., 2013). In homozygous twins discordantly infected with HCMV, those infected with the herpesvirus suffer more severe reactions to Flu infection (Brodin et al., 2015; Picarda and Benedict, 2018). There is gathering evidence that persistent infection with HCMV can impact on immune system function (Picarda and Benedict, 2018). Thus, the virus possesses numerous proteins that blunt immune response to infected cells and some of these activities could have general paracrine effects. Several additional resident viruses present in normal animals could also impact on the outcome pathogenic virus infections. So far the effects of the virome have mainly focused on their influence on the outcome of non-viral pathogens. For instance, an early report from the Virgin group noted that that mice latently infected with the herpesvirus HV68 or MCMV were more resistant to bacterial infection with *Listeria monocytogenes* and *Yersinia pestis* (Barton et al., 2007). These protective effects were attributed to systemically activated macrophages and upregulation of cytokines such as interferon-gamma. Additionally, mouse norovirus infection may increase the survival of mice challenged in the respiratory tract with *Pseudomonas aeruginosa*, an effect caused by a reduced IL-6 and TNF-alpha response to infection (Thépaut et al., 2015).

It is also curious to note that mice raised in pet stores (often referred to as a dirty environment) rather than in vivariums are infected with several viruses and other microbes and had antibodies to more agents than laboratory-raised animals (Beura et al., 2016; Masopust et al., 2017). The pet store mice also had more abundant naïve and memory T cells in lymphoid and non-lymphoid tissues (Beura et al., 2016; Masopust et al., 2017). Particularly striking was the high levels of tissue-resident memory cells in the tissues of dirty compared to lab-raised mice. Of interest, mice raised in the dirty state had immune cell response patterns that more resembled the human situation than occurred with laboratory-raised animals (Reese et al., 2016). Thus when performing pathogenesis and immune protection studies in mice using human viruses, perhaps we should be using pet store rather than laboratory-raised animals for such studies. In support of this idea, the Masopust group compared the response pattern to yellow fever virus vaccination in normal laboratory mice with those subjected to sequential infection with unrelated pathogens such as herpesviruses Flu and intestinal helminths. The dirty/co-infected mice compared to laboratory mice had enhanced baseline and vaccination responses measured by several assays and by metagenomic analysis had a response pattern resembling that observed in human adults frequently exposed to infections.

## The Influence of Previous and Concomitant Infections

The impact of a virus infection can be markedly affected by past or ongoing infection. The subject is a complex and the story is still unfolding. When different infections occur closely together the pattern of innate immune responsiveness to one or both agents critically affect the outcome. However, when infections are separated by several days, the response pattern of the adaptive immune response to the first infection also plays a crucial role. Older studies by the McKanass group in the early 1960s had observed that infection with one intracellular pathogen provided resistance for a few days to infection by an unrelated agent. The effect was attributed at that time when cellular immunology was still in its infancy, to non-specific protective effects of activated macrophages (Mackaness, 1962). More recently, the Chisari group showed that infection with LCMV caused reduced liver immunopathology caused by hepatitis B virus infection an effect also attributed to enhanced responses of innate aspects of immunity (Cavanaugh et al., 1998). With regard to the outcome of sequential infections with different viruses, the subject has been carefully studied for many years in mice models by the Selin and Welsh group (Welsh et al., 2010). Their studies have identified mechanistic circumstances which result in the second infection either being effectively controlled or to result in a tissue damaging immunopathological reaction that is more severe than would occur in a naïve infected animal. We shall discuss the highlights of their results later.

Closely spaced infections with different agents most commonly involve viral with non-viral infections. This happens, for example, with Flu which is usually accompanied by respiratory bacterial infections. When Flu becomes a severe clinical problem with even lethal results, secondary bacterial infections usually contribute to the severe tissue damage (Goka et al., 2015). Indeed, patients with severe Flu are normally treated with antibiotics and one assumes that this therapy is valuable. When coinfections with different viruses occurs, the outcome is affected by interference phenomena. The most common mechanisms of interference involve several types of interferon proteins which act to make infected cells antiviral and to influence the function of other cells, especially cells involved in immune reactions (Kumar et al., 2018). The functional effects include activation of immune cells, changes of signaling pathways and modulation of function via the induction of a large number of interferon-stimulated gene products. This topic has received many reviews (Dianzani, 1975; Otto, 2015). In instances where different viruses happen to infect the same cells, additional mechanisms of interference come into play. These so-called intrinsic interference phenomena, which was first studied with Newcastle disease virus (Marcus and Carver, 1967), include defective interfering particles, *trans-*acting proteases, non-specific double-stranded RNA and some others were recently reviewed in depth by Kumar et al. (2018). This review also lists many examples of co-infections along with their outcomes. One of the more notable consequences of two virus strains infecting the same cell is the production of viral recombinants which may have enhanced pathogenicity. This mechanism is at play when antigenic shift variants occur with influenza virus infection. The shift variants are often responsible for major Flu pandemics. Genetic reassortments also occur with other infections (Becher et al., 2001).

One major example from the veterinary world occurs when cows are persistently infected with bovine virus diarrhea (BVD) virus. This virus can recombine with other BVD strains, including vaccine strains, to generate highly lethal recombinants which causes a lethal mucosal disease in cattle (Becher et al., 2001). Intrinsic interference can also occur between unrelated viruses which may explain how enterovirus infection can inhibit the effectiveness of poliovirus vaccination (Becher et al., 2001).

The best studied examples where prior infections affect the outcome of a subsequent virus infection involves changes imposed on the adaptive immune responsive pattern that resulted from the first infection. This is often referred to as heterologous immunity (Welsh et al., 2010). In human disease situations, there are a few notable examples although actual mechanistic understanding is less forthcoming than is available from experimental situations with mouse models. A current relevant example involves the flaviviruses dengue and Zika. In dengue, where there are 4 major viral serotypes, some infected persons, usually children, develop a severe inflammatory reaction. This represents a cytokine storm and is called dengue hemorrhagic fever/dengue shock syndrome (DHF/DSS) (Halstead, 2007). The often lethal syndrome involves fever, hypotension, vascular leakage, thrombocytopenia and damage to several organs. Most of the time (but there are exceptions) DHF/DSS occurs in young persons who had already been infected with a different strain of dengue than the current infection. Some time ago Scott Halstead hypothesized that DHF/DSS represented the consequence of non-neutralizing antibody facilitated infection of inflammatory cells such as macrophages which mediate the pronounced inflammatory reaction (Halstead and O’Rourke, 1977). This was called antibody-induced enhancement (ADE). ADE still holds sway but other explanations have been advocated. These include the involvement of low avidity CD8 T cells which act to mediate immunopathology rather than controlling the infection (Mathew and Rothman, 2008).

More recently interest has focused on the relevance of heterologous immunity with the cross reactive flaviviruses dengue and Zika (Langerak et al., 2019). *In vitro* studies have provided evidence for ADE *in vitro* in both directions (Littaua et al., 1990; Bardina et al., 2017). However, the situation *in vivo* remains in debate although more convincing results show that pre-infection with Zika makes subsequent responses to dengue more consequential than the obverse situation where data are inconsistent (Langerak et al., 2019). Persons seropositive for dengue may be more likely to transmit Zika virus across the placenta to infect the fetus especially in late trimesters when placental transfer does not usually occur (Langerak et al., 2019). Prior infection with Zika can result in more severe lesions in persons subsequently infected with dengue, but this is by no means a regular occurrence and overt DHF/DSS is unusual (Langerak et al., 2019). The main relevance of unraveling whether or not heterologous severe responses occurs with double infections impacts on vaccine use. Thus, already a vaccine against the 4 strains of dengue has been withdrawn because of the increased incidence of DHF/DSS in some vaccines who were seronegative when vaccinated (Aguiar et al., 2016; Plotkin, 2019). A vaccine against Zika might also have adverse effects in some persons subsequently exposed to dengue. Similarly, dengue vaccines might cause more severe reactions when exposed to Zika as well as to some other cross-reacting flaviviruses such as West Nile virus (Bardina et al., 2017). A particular concern is that dengue vaccines could make Zika infected women more likely to transmit virus to the fetus.

Heterologous damaging immune reactions have been reported with other human viral diseases. One such example could be infectious mononucleosis (IM) occurring in young adults upon primary infection with the gamma herpesvirus Epstein Barr virus (EBV) (Aslan et al., 2017). Thus in HLA2 positive persons cross reactivity of CD8 T cells occurs between T cells that recognize epitopes from a Flu virus matrix protein and the protein BMLF1280 of EBV. The cross reactive T cells generated in response to Flu, which can be shown by *in vitro* studies to react with EBV, were proposed to be inadequate to control EBV infection, but instead contributed to inflammatory reactions upon EBV infection (Aslan et al., 2017). This concept of one virus inducing non-protective T cell responses that are expanded by the second infection which then mediate tissue damaging reactions was carefully studied by Selin and Welsh in mice using the cross-reacting arenaviruses LCMV and Pichinde virus and an unrelated virus Vaccinia (Selin et al., 1998). They focused on the numbers, specificity and avidity characteristics of the memory T cell repertoire induced by primary and secondary infection. Of interest, the nature of the memory response to the first infection impacted on the type of response made to the subsequent cross reacting pathogen. The T cell repertoire responding to the first infection consisted of multiple different specificities and binding efficiencies. Exposure to the heterologous second infection served to expand only the cross-reactive cells, many of which had low avidity and produced minimal protective cytokines such as interferon-gamma. Consequently, the second infection was not promptly controlled. However, these non-protective T cells could help orchestrate a tissue damaging inflammatory reaction.

Other interpretations for the untoward outcome of heterologous infections includes effects on the balance of several immune components preferentially expanded by the second infection. Thus, if the response is non-inflammatory, it could be explained by the dominance of regulatory aspects of immunity expanded by the second infection. These include regulatory T cells and anti-inflammatory cytokines such as IL-10 and TGF-beta. Inflammatory responses would accrue from the preferential expansion of Th1 and Th17 cells, M1 macrophages and cells producing IL-6, TGF-beta, and other inflammatory molecules. This concept has been discussed in detail in other reviews (Gil et al., 2015; Souquette and Thomas, 2018).

In this current world where measles has re-emerged as a problem infection and we still suffer the onslaughts of HIV infection, it is pertinent to point out that some virus infections are highly suppressive to the immune system such that multiple infections, including several viruses that in normal persons are well controlled become significant pathogens. Measles virus is one of the most immunosuppressive of all infections and persons with clinical measles readily suffer problems with other infections, some of which can be lethal (Griffin et al., 2012). HIV infection is always persistent and it takes many months in untreated persons for them to become markedly immune suppressed. Once this happens, normally non-troubling infections by herpesviruses and other agents become problematic. For example, the normally inapparent HCMV infection can cause a blinding retinitis (Alan, 1988; Salzberger et al., 2005). In addition, the usually silent Kaposi sarcoma herpesvirus infection can cause Kaposi sarcomas and primary or reactivation of HSV can result in severe dermal lesions which can disseminate to the brain and cause encephalitis (Sepkowitz, 2001; Rezaee et al., 2006). This topic has received several reviews including one by our own group (Sehrawat et al., 2018).

## How Can We Reset Immune Response Patterns to Minimize Tissue Damaging Responses to Virus Infections?

We have argued that in many instances where a viral infection causes overt tissue damage rather than being controlled without notice happens because of some defect or imbalance in the host response to the infection. Accordingly, controlling such events would require immune replacement or rebalance of the immune response pattern. The traditional approach to control virus induced lesions is to combine specific antiviral drugs or monoclonal antibodies (few viruses can be controlled in this way) along with counteracting the inflammatory response. The latter is commonly achieved with steroids, non-steroidal anti-inflammatory drugs and occasionally other approaches such as immunosuppressants, specialized pro-resolving mediators, and some other novel approaches (Spite et al., 2014; Bhela and Rouse, 2018). There is a need for additional approaches particularly to control inflammatory lesions associated with persistent viral infections. We shall briefly discuss three approaches we consider to be promising.

The general approach that currently enjoys the most attention is checkpoint blockade. The strategy is being widely used to improve immunity to several cancers (O’Sullivan and Pearce, 2015). Its success depends on using mAb that engage and inhibit costimulatory molecules expressed at the surface of T cells which when bound to their normal ligand act to inhibit the protective function of T cells. The popular parlance is to describe the inhibited T cells is exhaustion (Roy et al., 2018). Such exhausted T cells were first noted in the chronic virus infection LCMV (Barber et al., 2006), but the phenomenon has now been observed in several other infections (Wherry, 2011). Indeed, there is a tendency to exaggerate the relevance of exhaustion as explaining the failure to control chronic tissue damaging infections. In the best studied system, which remains LCMV, exhaustion appears real and when it is reversed, as can be done with mAb against several molecules that include PD1, PDL1, TIM3, CTLA4, LAG-3, and perhaps others the course of inflammatory events can be inhibited and the virus better controlled (Barber et al., 2006; Roy et al., 2018). Many entertain the idea that reversing exhaustion could be useful to help control HIV, HSV, HCV, and hepatitis B virus (Saeidi et al., 2018). For instance, reversing the function of putative exhausted T cells was shown to reduce the severity of lesions following herpesvirus recurrences (Allen et al., 2011). Moreover, the approach was advocated as a potential means to facilitate the efficacy of therapeutic vaccines against HSV, HIV as well as a way to prevent human papilloma induced cancers in already infected persons (Palefsky, 2009). The value of the approach was recently reported to protect against recurrent ocular HSV lesions (Roy et al., 2018). The use of checkpoint blockade to control chronic virus disease and potentiate vaccine merits further evaluation paying attention to side effect than might occur such as increasing the consequences of some inflammatory diseases (Johnson et al., 2018).

A second viable approach that might prove useful to change the course of tissue damaging virus infections is to manipulate miRNA expression. Thus several reports indicate that the expression of either host or viral miRNA can influence aspects of viral pathogenesis (Gottwein and Cullen, 2008; Ressel et al., 2019). For example, in a HSV model of immunopathology mice that lacked the expression of miR155, because of gene knockout, had markedly reduced lesions of herpetic stromal keratitis (SK) (Bhela et al., 2014). Similarly, inhibition of miR155 using an antagomer approach also reduced lesion severity. The effects occurred because miR155 is necessary for the function of proinflammatory Th1 and Th17 T cells that orchestrate the ocular lesions. A similar outcome was also observed in a model of inflammatory encephalitis caused by Japanese encephalitis virus (JEV) (Thounaojam et al., 2014). In this JEV model, knockdown of miR155 with antagomers resulted in less encephalitis (Thounaojam et al., 2014). Modulation of other miRNA such as miR-15b and 19-3b, both involved in inflammatory responses, were also studied in the JEV system. In such studies therapy with antagomers diminished lesions and levels of the inflammatory mediators IL-1β, IL-6, and CCL2 were decreased apparently a consequence of reduced RIG-1 signaling (Zhu et al., 2015; Ashraf et al., 2016).

Other miRNA whose modulated expression could impact on viral lesions include those critical for angiogenesis such as miR132 (Mulik et al., 2012), those such as miR-10a, miR-146a, and miR-17 which might act by enhancing T_reg_ function, as well as those that can promote the resolution of inflammation by regulating specialized pro-resolving mediators (Mulik et al., 2013). miRNAs can also control the production of pro and anti-inflammatory cytokines from innate immune cells (Tahamtan et al., 2018). In this regard our own unpublished data indicates that miR-142 acts as an anti-inflammatory miRNA by potentially targeting proinflammatory cytokine IL-6, thereby preventing an early onset of SK.

With some viruses their replication and consequent tissue damage is facilitated by host miRNA species. This is true for HCV which uses host miR122 to facilitate its replication (Janssen et al., 2013). In fact, drugs which target miR122 such as the antagomer miravirsen which inhibits HCV replication are currently in clinical trials.

We can surmise that manipulating disease associated miRNAs hold promise but there are many problems to solve. These include targeting to appropriate sites and realizing that each miRNA may have additional functions giving rise to unwanted off-target effects.

A so far poorly explored, but potentially valuable approach to make responses to virus infections less tissue damaging is to take advantage of the fact that cells of the immune system differ in their metabolism. Thus, as lucidly reviewed by O’Neill et al. (2016), cells of the immune system employ 6 major metabolic pathways to support their functions, but these are used differentially by cells that participate in immune processes. Accordingly, drugs that target metabolic pathways to increase or decrease their activity should act differentially on the various cells involved in immune defense or tissue damage. Using drugs that target metabolic pathways was pioneered in the cancer field exploiting the fact that cells involved in reacting to tumors rely on different pathways to support their energy and other metabolic requirements (Pearce et al., 2013). For example, pro-inflammatory T cell subsets (Th1, Th17), inflammatory M1 macrophages, activated neutrophils (Loftus and Finlay, 2016) rely mainly on aerobic glycolysis (Warburg Effect) and the pentose phosphate pathway to provide their energy. In contrast, the counter inflammatory components such as T_reg_ and M2 macrophages obtain their energy using oxidative phosphorylation and fatty acid oxidation (O’Neill and Hardie, 2013; Galván-Peña and O’Neill, 2014). Limited studies have so far been done using metabolic pathway inhibitor drugs to modulate viral pathogenesis. However, one study showed that if oxidative glycolysis was inhibited using 2-Deoxy-D-glucose (2DG) in a model of viral immunopathology then lesions were markedly diminished (Varanasi et al., 2017). The outcome was explained by a selective inhibitory effect on proinflammatory CD4 T cells permitting uninhibited regulatory T cells to remain active (Varanasi et al., 2017). The effects of 2DG has also been studied in a rhinovirus model of lung damage. Here too 2DG resulted in reduced lesions (Gualdoni et al., 2018).

Using 2DG during a viral infection can result in untoward effects especially in situations where virus replication in non-immune cells is the primary cause of pathology. For example, in the HSV model in mice, therapy with 2DG in the acute phase of infection resulted in suppression of the protective aspects of defense. This allowed the virus to spread to the brain to cause lethal encephalitis (Varanasi et al., 2017). In an influenza model too, 2DG therapy in the early phases of infection resulted in higher levels of mortality (Wang et al., 2016).

Additional metabolic pathways such as amino acid metabolism are beginning to be studied for effects on the outcome of viral infections. In Sindbis virus infected mice, for example, treatment of mice with the drug 6-Diazo-5-oxo-L-norleucine (DON), which inhibits glutamine metabolism, diminished the immunoinflammatory lesions in the brain and spinal cord which result in paralysis and mortality (Manivannan et al., 2016; Baxter et al., 2017). Changing the fatty acid synthesis metabolic pathway as can be done using the drug PF-05175157 has also led to reduced viral immunoinflammatory lesions caused by West Nile virus infection (Jiménez de Oya et al., 2019). Modulating different metabolic pathways could also turn out to be a viable approach to influence the expression of some persistent infections such as herpesvirus latency. Examples include suppressing viral reactivation by manipulating glutamine availability (Wang et al., 2017) and modulating fatty acid oxidation which can influence the breakdown of latency *in vitro*.

## Conclusion

We are constantly exposed to viruses but few of them have the intrinsic pathogenicity to cause significant damage to normal hosts. We have reviewed the circumstances of infection and the status of the host which does favor tissue damage. Understanding the details of virus-host interaction which leads to disease is expected to result in developing novel approaches to prevent the untoward effects of infections.

## Author Contributions

BR conceptualized the manuscript. BR, DS, and EB wrote the manuscript.

## Conflict of Interest

The authors declare that the research was conducted in the absence of any commercial or financial relationships that could be construed as a potential conflict of interest.
